# Complete genome determination of a European *Bombyx mori* nucleopolyhedrovirus (BmNPV) strain isolated in the French Cevennes

**DOI:** 10.1128/mra.01159-24

**Published:** 2025-06-10

**Authors:** Christophe Loizel, Théophile Grébert

**Affiliations:** 1 Nevezyne, Pôle Biotechnologique, Ploufragan, France; 2 Scilicium, Scilicium, Rennes, France; Katholieke Universiteit Leuven, Leuven, Belgium

**Keywords:** *Bombyx mori *nucleopolyhedrovirus, baculovirus, silkworm

## Abstract

The Bombyx mori nucleopolyhedrovirus (BmNPV) is a critical pathogen in sericulture; yet, it also holds great promise for the bioproduction of recombinant proteins. Here, we report the characterization of a highly pathogenic strain from the French Cevennes region. Phylogenetic analyses reveal that this strain is related to a Brazilian BmNPV strain.

## ANNOUNCEMENT

BmNPV, belonging to the *Baculoviridae* family, infects silkworms (*Bombyx mori*) selectively and has been exploited for recombinant protein production due to its ability to express high levels of foreign proteins in infected cells or silkworms (1–3).

Sericulture has a long-standing tradition in France, particularly in the Cévennes region. French sericulture peaked in the 19th century when silk was a highly valued luxury item, but saw a steep decline in the early 20th century, notably due to diseases affecting silkworms. In recent decades, there has been renewed interest in silk production. A severe grasserie epidemic broke out in the French Cévennes in 2022, which caused up to 100% mortality among certain breeders (Personal communication from the Cévennes silkworm’s breeders). During this outbreak, *Bombyx mori* larvae exhibiting symptoms of BmNPV infection were collected alive from Cévennes farms. The virus was isolated from these naturally infected larvae and propagated and purified using BmN cells (4).

Genomic DNA was extracted by alkaline lysis of virions as described (5), column-purified (QIAamp DNA Blood Kits, Qiagen), and sequenced using paired-end reads (2 × 150 bp) on an Illumina NovaSeq 6000 platform (Eurofins Genomics). In all, 9,650,470 reads (mean read length 146 bp) were preprocessed using fastp (6). The genome was assembled using Unicycler (7), and visualization of the assembly graph revealed a circular contig of 127 kb corresponding to the expected structure and size of BmNPV genomes. The assembled genome was annotated using Prokka (8) with publicly available BmNPV sequences as references and manually curated. Comparative genomics was performed using Roary (9) and visualized with the pyCirclize Python library. Phylogenetic reconstruction was done using FastTree (6) based on 47 core genes identified by Roary (option -n) and aligned using MAFFT (10). All tools were used with default settings, except stated otherwise. GC content and genome nucleotide similarity were computed using SnapGene software V8.0.

**Fig 1:**
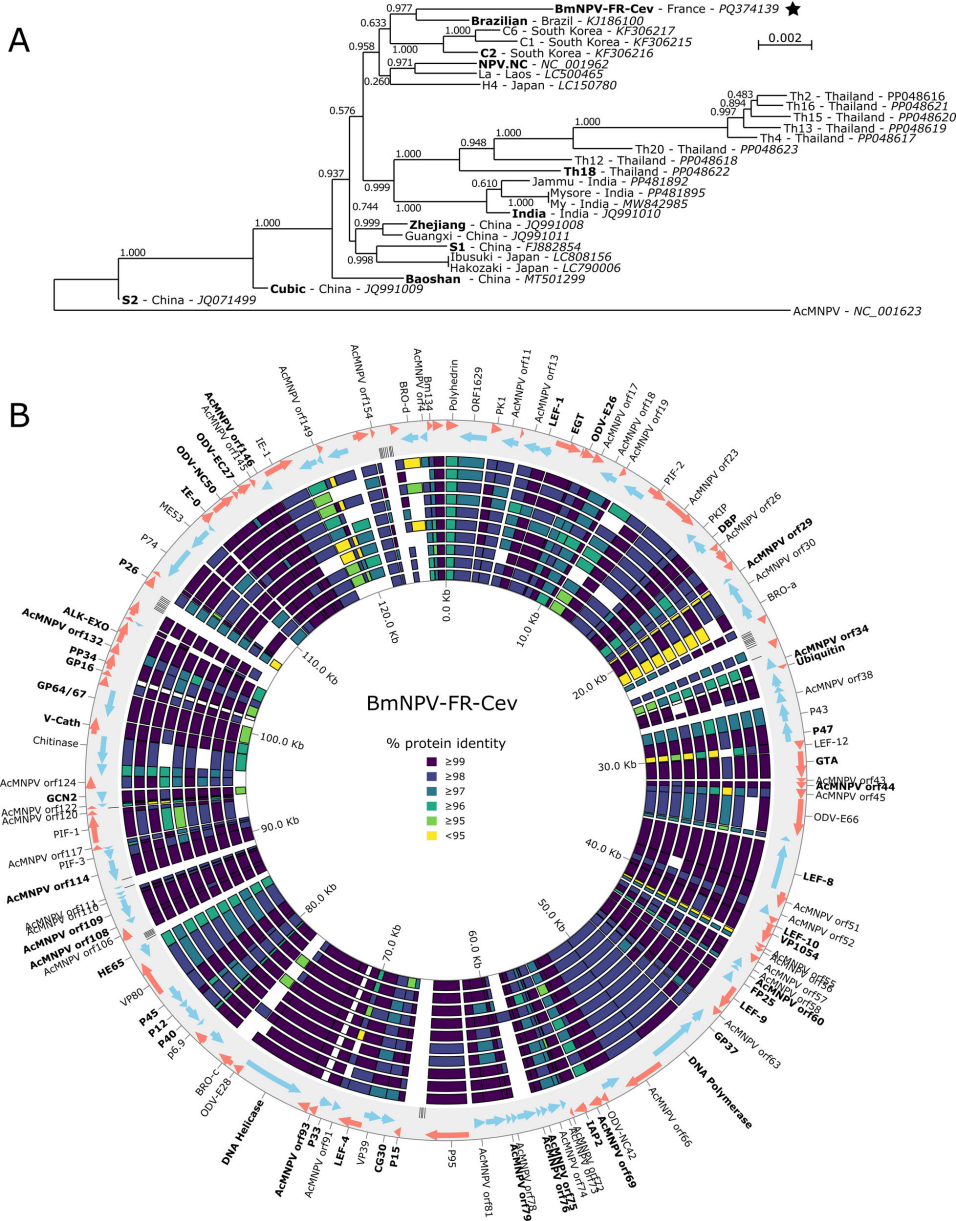
Comparison of BmNPV-FR-Cev with other sequenced BmNPV strains. (**A**) Phylogenetic tree obtained on an alignment of 47 core genes representing a total of 42,595 nucleotides using FastTree. BmNPV-FR-Cev is marked with a star, numbers represent FastTree local branch support. For each strain, the country of isolation (when available) and its GenBank accession number are indicated. (**B**) Comparison of genome composition and protein identity with 10 sequenced BmNPV strains. Predicted genes in BmNPV-FR-Cev are represented on the outer circle. Each of the 10 inner circles represents the comparison to each strain highlighted in (**A**), ordered from the phylogenetically closest on the outside to the most distant on the inside: Brazilian, C2, NPV.NC, Th18, India, Zhejiang, S1, Baoshan, Cubic, S2. Genes are colored according to the protein identity between BmNPV-FR-Cev and the compared strain, and genes not detected in the compared strains are left blank. The names of the core genes identified by Roary and used for (**A**) are indicated in bold.

The coding-complete genome of the BmNPV strain from the French Cévennes (named BmNPV-FR-Cev) is composed of 127,052 base pairs (G + C content of 40.28%) coding for 136 predicted genes (11). Phylogenetic analysis placed BmNPV-FR-Cev closest to the Brazilian strain of BmNPV (12), forming a well-supported clade with other strains, including C1, C2, C6, NPV-NC, La, and H4 (Fig. 1A). The synteny, or gene order, of the BmNPV-FR-Cev strain is preserved compared to other sequenced BmNPV strains, demonstrating a high degree of genetic similarity (Fig. 1B). Whole-genome comparisons revealed that BmNPV-FR-Cev shares 98.63% nucleotide identity with the Cubic strain (13), 98.69% with the Hakozaki strain (14), and 98.59% with Bombyx mandarina NPV (BomaNPV) S2 (15). Notably, the genome only carries three bro genes (bro-a, bro-c, and bro-d, Fig. 1B). In addition, BmNPV-FR-Cev features the eight homologous repeat (hr) regions (hr1, hr2L, hr2R, hr3, hr4a, hr4b, hr4c, and hr5), which are multifunctional elements crucial for viral replication, transcription, and recombination.

In summary, the BmNPV strain isolated from the French Cévennes demonstrates high virulence, making it a promising candidate for an infectious vector for bioproduction.

## Data Availability

The genome of BmNPV-FR-Cev has been deposited in GenBank under the accession no. PQ374139, https://www.ncbi.nlm.nih.gov/nuccore/PQ374139. The raw reads are accessible on NCBI’s SRA under the accession PRJNA1244288 (https://www.ncbi.nlm.nih.gov/sra/PRJNA1244288). pyCirclize : https://moshi4.github.io/pyCirclize/ snapgene : https://www.snapgene.com/.
